# Hospitals, the Sick Poor, and All England

**Published:** 1887-01-29

**Authors:** Henry C. Burdett


### Hospitals: The Sick Poor and All England.

By Henry C. Burdett.

The rapid growth of the population of Great Britain in recent years has caused a corresponding increase in the number and variety of those cases of sickness among the poor, which can only be adequately treated in hospitals. Along with the increase of these cases there has been a similar growth in the provision for meeting their necessities. But whilst the sick abound and the hospitals are numerous, there is often experienced very great difficulty in finding out the *right hospital* for the *right patient* at the *right time.* Hence arises much unnecessary suffering, with loss of time and money, and sometimes even the loss of life itself. These circumstances have for a long time forced themselves upon hospital managers, but there has appeared to be no general method applicable to all cases, which would at once and entirely put an end to the difficulties.

*The Hospitals Association* was formed some years ago with the view of becoming a central meeting-ground for all hospitals on the one hand, and for all on the other hand who might desire to come into relationship with hospitals, either as patients or in any other capacity. To a considerable extent these objects have been gained, and now, by means of the Association, it is possible to place a given patient in a given hospital with little or no loss of time, and also to gain any kind of knowledge which may be desired concerning almost every hospital in the United Kingdom or in the world.

The resources of the Association have recently received such a degree of development, that the objects which formerly were but partially and incompletely carried out, will in a short time be perfectly accomplished, and all these resources will then be placed at the disposal of every member of the Association, and every institution which may be now or hereafter affiliated with it.

It is desired to bring every parish or congregation, and even the remotest villages and hamlets, by *affiliation*, into immediate and permanent relationship with the hospital system of the United Kingdom, and with the Hospitals Association as the centre of the system. By this means every person needing the peculiar treatment which hospitals alone can give will be enabled to obtain, if eligible, without inconvenience or loss of time, the highest degree of medical and surgical skill which the science of the time affords.

If the efforts of the Association be adequately seconded, it is hoped to establish by its agency a fully-equipped ambulance system for each large centre of population; and, by arrangements made with the various railway companies, to secure the cheap and easy removal to the nearest hospital of persons living in remote and outlying districts.

In order to the complete carrying out of these beneficent objects, the Association has prepared a scheme which is hereby submitted to the parish clergy and the ministers of congregations, and also to all other persons who may be interested in the care and cure of the sick poor. The scheme is as follows:—

I. Each parish, congregation, village, and hamlet is invited, through its clergyman, minister, or other representative, to affiliate itself to the Hospitals Association by the formation of a "Hospital Society." In either case the annual subscription will be one guinea. Individuals desiring to become *Life Members* can do so by payment, in one sum of ten guineas. These payments will secure all the advantages detailed below, together with the weekly newspaper, The Hospital. It is hoped that wherever it is practicable a "Hospital Society" will be formed, consisting of the clergyman or minister as the president, the local doctor or doctors, and two honorary secretaries, one of whom should be a lady. There can be little doubt that the poorer the parish or congregation the greater would be the advantages derived from affiliation. With the view of bringing these proposals more fully under the attention of those actively interested in the welfare of the sick poor, a congress will be held in May next, under the presidency of Sir Andrew Clark, Bart., M.D., to explain, consider, and finally consolidate the work now begun.

II. The Hospitals Association will be an *Immediate Aid Society*, and its central office will be in London. The Secretary will give prompt assistance in the placing of difficult and other cases in those hospitals which may be most suitable for them. Any clergyman or minister of an affiliated congregation, having difficult cases for hospital treatment, instead of making applications in all directions, and often fruitlessly, will at once communicate with the Secretary at the Central Office, and his difficulty will be at end.

III. The office will be a *Bureau of Information*, and a *Centre of Reference*. It will give all possible facilities for gaining the completest knowledge of any and every hospital in the kingdom, and of hospital affairs generally. It will arrange for lectures and illustrations of hospital life for the winter months, and provide trained and eminent lecturers. It will give aid and advice in the management of public meetings, and co-operate in obtaining men of standing and ability as speakers on specially important occasions.

IV. The new Journal of the Association, The Hospital, which is published weekly, will be sent post free every week to each subscribing member of the Association, or affiliated parish, or congregation. The Hospital will contain the latest, most reliable, and most interesting information concerning hospitals and kindred charities, as well as instructive articles on medical and sanitary questions and related topics.

V. Facilities will be offered whereby members of the Association, and of affiliated Societies, may from time to time visit and inspect the general and special hospitals and other institutions of a like kind, thereby obtaining at first hand whatever knowledge of the subject they may wish or require.

Such is the plan which the Hospitals Association cordially commends to clergymen and ministers of religion generally, and to all other persons who are concerned for the well-being of the sick poor. It is conceived in the interests of the poor, and of those who have the care of them. It is easy to see, however, that not only will it be of inestimable service to those for whom it is primarily intended, but that the hospitals and their work will thus be brought into immediate practical relation with every parish in the kingdom, and will so become known, valued, and supported as they urgently need and justly deserve.

There can be no question that if the scheme becomes popular, as it is hoped, much unnecessary suffering will be prevented, much waste of time avoided, and much hostile criticism of hospitals, their work and their methods, brought to an end.

Applications for membership, or for further information, should be made to the Secretary, Hospitals Association, Norfolk House, Norfolk Street, Strand, London, W.C.

